# Tick-Borne Viruses and Biological Processes at the Tick-Host-Virus Interface

**DOI:** 10.3389/fcimb.2017.00339

**Published:** 2017-07-26

**Authors:** Mária Kazimírová, Saravanan Thangamani, Pavlína Bartíková, Meghan Hermance, Viera Holíková, Iveta Štibrániová, Patricia A. Nuttall

**Affiliations:** ^1^Department of Medical Zoology, Institute of Zoology, Slovak Academy of Sciences Bratislava, Slovakia; ^2^Department of Pathology, University of Texas Medical Branch Galveston, TX, United States; ^3^Institute for Human Infections and Immunity, University of Texas Medical Branch Galveston, TX, United States; ^4^Center for Tropical Diseases, University of Texas Medical Branch Galveston, TX, United States; ^5^Biomedical Research Center, Institute of Virology, Slovak Academy of Sciences Bratislava, Slovakia; ^6^Department of Zoology, University of Oxford Oxford, United Kingdom; ^7^Centre for Ecology and Hydrology Wallingford, United Kingdom

**Keywords:** tick, arbovirus, transmission, skin, immunomodulation, vaccines

## Abstract

Ticks are efficient vectors of arboviruses, although less than 10% of tick species are known to be virus vectors. Most tick-borne viruses (TBV) are RNA viruses some of which cause serious diseases in humans and animals world-wide. Several TBV impacting human or domesticated animal health have been found to emerge or re-emerge recently. In order to survive in nature, TBV must infect and replicate in both vertebrate and tick cells, representing very different physiological environments. Information on molecular mechanisms that allow TBV to switch between infecting and replicating in tick and vertebrate cells is scarce. In general, ticks succeed in completing their blood meal thanks to a plethora of biologically active molecules in their saliva that counteract and modulate different arms of the host defense responses (haemostasis, inflammation, innate and acquired immunity, and wound healing). The transmission of TBV occurs primarily during tick feeding and is a complex process, known to be promoted by tick saliva constituents. However, the underlying molecular mechanisms of TBV transmission are poorly understood. Immunomodulatory properties of tick saliva helping overcome the first line of defense to injury and early interactions at the tick-host skin interface appear to be essential in successful TBV transmission and infection of susceptible vertebrate hosts. The local host skin site of tick attachment, modulated by tick saliva, is an important focus of virus replication. Immunomodulation of the tick attachment site also promotes co-feeding transmission of viruses from infected to non-infected ticks in the absence of host viraemia (non-viraemic transmission). Future research should be aimed at identification of the key tick salivary molecules promoting virus transmission, and a molecular description of tick-host-virus interactions and of tick-mediated skin immunomodulation. Such insights will enable the rationale design of anti-tick vaccines that protect against disease caused by tick-borne viruses.

## Introduction

Ticks are familiar to most people world-wide. They have accompanied humans through their long history, known as blood-sucking creatures that decimate livestock. However, since the pioneering work of Smith and Kilbourne on Texas fever of cattle in 1893, followed by Rickett's discovery of the pathogen causing Rocky Mountain spotted fever transmitted by *Dermacentor andersoni* in 1907, ticks have been identified as vectors of a huge range of viral, bacterial, and protozoan agents of diseases, and have become a major focus of medical and veterinary research (Nicholson et al., 2009; Sonenshine and Roe, 2014). It is now recognized that ticks surpass all other arthropods in the variety of transmitted infectious agents involving nematodes, fungi, protozoa, bacteria, and viruses. Tick-borne viral diseases have significant and increasing medical and veterinary impact due to the geographical spread of the vectors and outbreaks in new regions as a result of changes in global socio-economic and climatic conditions, and lack of efficient control measures (Estrada-Peña et al., 2013; Nuttall, 2014; Vayssier-Taussat et al., 2015; Brackney and Armstrong, 2016). Here, we consider the many viruses transmitted by ticks, the reasons why ticks are such efficient and effective vectors of viruses, and future directions for research.

## Tick-borne viruses

Tick-borne viruses (TBV) or “tiboviruses” (Hubálek and Rudolf, 2012) comprise a diverse group of viruses circulating between ticks and vertebrate hosts, thriving in two extremely different environments: the homeostatic environment of the vertebrate host and the dramatically changing environment of ticks. TBV and ticks have evolved together, resulting in a complex relationship in which the virus life cycle is perfectly coordinated with the tick's feeding cycle, and the tick can harbor the virus for prolonged periods without affecting its biology. Considering their unique characteristics, ticks are believed to shape the evolution of TBV (Nuttall and Labuda, 2003; Kuno and Chang, 2005).

The initial discoveries of arboviruses such as yellow fever virus (1928) (mosquito-borne), and Nairobi sheep disease virus (1910) and louping ill virus (LIV) (1929) (tick-borne), opened the floodgates for the discovery of over 500 arboviruses during the ensuing years (Bichaud et al., 2014). At least 160 named viruses are tick-borne, of which about 50 are recognized or probable “virus species” (Nuttall, 2014). Taxonomically, TBV comprise a heterogenous group of vertebrate viruses classified into one DNA viral family, *Asfarviridae*, and eight RNA viral families: *Flaviviridae, Orthomyxoviridae, Reoviridae, Rhabdoviridae*, the newly recognized *Nyamiviridae* (order *Mononegavirales*), and the families *Nairoviridae, Phenuiviridae*, and *Peribunyaviridae* in the new order, *Bunyavirales* (Table 1).

**Table 1:** Classification of tick-borne RNA viruses including recently described species, with indication of viruses causing major diseases of humans and domesticated animals.

**Family**	***Flaviridae*** **(ssRNA** +**)**			***Reoviridae*** **(dsRNA)**	
**Subfamily/group**	**Mammalian tick-borne flavivirus**	**Seabird tick-borne flavivirus**	**Kadam tick-borne flavivirus**	***Sedoreovirinae***	***Spinareovirinae***
**Genus**	***Flavivirus***	***Flavivirus***	***Flavivirus***	***Orbivirus***	***Coltivirus***
Species	Tick-borne encephalitis virus (TBEV)^††^	Tyuleniy virus	Kadam virus	Chenuda virus	Colorado tick fever virus
	Louping ill virus (LIV)^†^	Meaban virus		Chobar Gorge virus	Eyach virus
	Langat virus	Saumarez Reef virus		Great Island virus	
	Powassan virus (POWV)^††^			Wad Medani virus	
	Kyasanur Forest disease virus (KFDV)^††^			St Croix River virus	
	Omsk hemorrhagic fever virus				
	Gadgets Gully virus				
	Royal Farm virus				
Unassigned				Lake Clarendon virus	
				Matucare virus	
**Family**	***Orthomyxoviridae*** **(segmented ssRNA-)**		***Rhabdoviridae*** **(ssRNA-)**		***Nyamiviridae*** **(ssRNA-)**
**Genus**	***Thogotovirus***	***Quaranjavirus***	***Vesiculovirus***	***Ledantevirus***	***Nyavirus***
Species	Thogoto virus	Quaranfil virus	Isfahan vesiculovirus	Barur ledantevirus	Nyamanini virus
	Dhori virus	Johnston Atoll virus		Kern Canyon ledantevirus	Midway nyavirus
				Kolente ledantevirus^*^	Sierra Nevada nyavirus
				Yongjia ledantevirus^*^	
					
Unassigned			Connecticut virus		
			New Minto virus		
			Sawgrass virus		
**Order**	***Bunyavirales*** **(segmented ssRNA-)**				
**Family**	***Nairoviridae***		***Peribunyaviridae***	***Phenuiviridae***	
**Genus**	***Orthonairovirus***	**Putative** ***Nairoviruses***	***Orthobunyavirus***	***Phlebovirus***	
Species	Burana orthonairovirus	Artashat virus	Bakau orthobunyavirus	Severe fever with thrombocytopenia syndrome phlebovirus (SFTSV)^††^^*^	
	Crimean-Congo hemorrhagic fever orthonairovirus (CCHFV)^††^	Burana virus	Estero Real orthobunyavirus	Uukuniemi phlebovirus	
	Dera Ghazi Khan orthonairovirus	Chim virus	Tete orthobunyavirus		
	Dugbe orthonairovirus	Geran virus			
	Hazara orthonairovirus	Nàyǔn tick virus^*^			
	Hughes orthonairovirus	South Bay virus^*^			
	Keterah orthonairovirus	Tamdy virus			
	Nairobi sheep disease virus orthonairovirus (NSDV)^††^				
	Qalyub nairovirus				
	Sakhalin nairovirus				

†† *Human pathogens and their principal vector ticks: TBEV—Ixodes persulcatus, I. ricinus; KFDV—Haemaphysalis spinigera; POWV—Ixodes scapularis; SFTSV—Haemaphysalis longicornis; CCHFV—Hyalomma spp*.

† *Non-human pathogens and their principal vector ticks: LIV—Ixodes ricinus; NSDV—Rhipicephalus appendiculatus*.

* *Recently described species*.

The occurrence of TBV in different viral families suggests that their tick-borne mode of transmission evolved independently at least seven times (Nuttall, 2014). Almost 25% of TBV are associated with disease and all TBV pathogenic to humans are zoonotic. At present, more than 16 specific tick-borne diseases (TBD) of humans and 19 TBD of livestock and companion animals have been described (Nicholson et al., 2009; Sonenshine and Roe, 2014). Several TBV cause serious human or animal diseases, such as CNS disease (meningitis, meningoencephalitis, or encephalomyelitis), or haemorrhagic disease (Table 1). Others are less serious or sporadically reported, and most are without known medical or veterinary significance. Certain viral diseases of feral vertebrates as well as domestic animals and even humans may pass unnoticed or are misdiagnosed, and eventually they may appear as emerging diseases (Dörrbecker et al., 2010; Hubálek and Rudolf, 2012).

In recent decades, a number of recognized TBV, mainly those belonging to the tick-borne encephalitis virus (TBEV) serocomplex, have emerged or re-emerged, and/or spread, posing an increasing threat to human and animal health. For example, a rise in the incidence of human infections caused by Powassan virus (POWV) in the USA (Hermance and Thangamani, 2017), the spread of TBEV into new geographic areas, and the emergence of new viruses such as Alkhurma virus, a subtype of Kyasanur forest disease virus (KFDV) (Charrel et al., 2001), and Deer tick virus, a subtype of POWV (Pugliese et al., 2007; Robertson et al., 2009; Hermance and Thangamani, 2017) have been reported. The latest emerging TBD, caused by Bourbon virus (*Thogoto virus, Orthomyxoviridae*), was reported in Kansas in 2014 (Kosoy et al., 2015).

While new TBV are being discovered, unclassified viruses are being allocated to genera or families thanks to improvements in molecular technologies (Table 1). The most notable changes have occurred in families *Bunyaviridae* and *Rhabdoviridae*. The *Bunyaviridae* has been revised and elevated to the order *Bunyavirales* comprising 9 families and 13 genera (Briese et al., 2016; Junglen, 2016; Walker et al., 2016b). TBVs are included in three families—*Nairoviridae, Phenuiviridae*, and *Peribunyaviridae*. Except for the most medically important member, Crimean-Congo haemorrhagic fever virus (CCHFV), the genus *Orthonairovirus* of the *Nairoviridae* comprises 11 other species, 9 of which are TBVs (Table 1) (Kuhn et al., 2016a,b; Walker et al., 2015, 2016b). The most recent emerging human disease causing significant mortality (up to 30% of cases) is caused by Severe fever with thrombocytopenia syndrome virus (SFTSV), a new member of the *Phlebovirus* genus (*Phenuiviridae*) first reported in China (Xu et al., 2011; Yu et al., 2011; Zhang et al., 2011). Recently, a new virus closely related to SFTSV named Heartland virus was isolated from severely febrile patients in the USA (McMullan et al., 2012) and from field collected ticks (Savage et al., 2013). Moreover, phylogenetic and serological analyses revealed that Bhanja virus and Palma virus (previously unassigned to a genus) are closely related to both SFTVS and Heartland virus (Dilcher et al., 2012; Matsuno et al., 2013).

Within the *Rhabdoviridae* family, a new genus, *Ledantevirus*, comprises 14 new species, four of which are TBVs (Blasdell et al., 2015; Walker et al., 2016a) (Table 1). In recent years, further novel rhabdoviruses have been identified from various animal species, but so far transmission by ticks have been confirmed only for a few of them, e.g., Kolente virus (Ghedin et al., 2013) and Yongjia tick virus 2 (YTV-2) (Li et al., 2015). For Long Island tick rhabdovirus (Tokarz et al., 2014) and Zahedan virus (Dilcher et al., 2015) transmission by ticks has yet to be confirmed.

Recent studies suggest that besides the hitherto only recognized DNA containing arbovirus, African swine fever virus (ASFV) (*Asfarviridae*, genus *Asfivirus*), transmitted by soft ticks, other DNA viruses may be transmitted by ticks. Lumpy skin disease virus (LSDV, *Capripoxvirus, Poxviridae*), the cause of skin disease in cattle, is transmitted by blood-feeding insects such as mosquitoes and stable flies (Carn and Kitching, 1995; Chihota et al., 2001, 2003), but recent studies indicate the potential for biological transmission of LSDV by *Amblyomma* and *Rhipicephalus* ticks (Tuppurainen et al., 2011, 2013a,b; Lubinga et al., 2013, 2014). Another DNA virus potentially transmitted by ticks is Murid herpesvirus 4 (MuHV 4) strain 68 (MHV-68), which has been detected in field collected ixodid ticks (Ficová et al., 2011; Kúdelová et al., 2015; Vrbová et al., 2016). Could this be the cause of the next emerging tick-borne virus disease?

## Ticks as vectors of viruses

Ticks have evolved several unique features - such as their prolonged life-span and complex development, haematophagy in all post-embryonic life stages, long feeding periods, and blood digestion within midgut cells—that contribute to their success as vectors of viruses (Sonenshine et al., 2002; Nuttall and Labuda, 2003). Once ticks acquire a virus, they usually remain infected for the rest of their life. Due to their exceptional longevity, ticks act as excellent reservoirs of TBV, carrying viruses over months or even years, and maintaining them transstadially from one developmental stage to the next prior to transmission to a vertebrate host (Nuttall et al., 1994; Nuttall and Labuda, 2003; Turell, 2015). TBV persistence in a tick population can also be ensured through transmission from infected females via eggs to their progeny, although the rates of transovarial transmission of TBV in nature appear to be low (Nuttall et al., 1994; Kuno and Chang, 2005).

Isolation of a virus (especially from an engorged tick), or detection of the presence of viral RNA or DNA in a tick, do not necessarily prove that a particular tick species is a competent vector of the virus (Nuttall, 2009). To determine vector competence, the following parameters have to be fulfilled: (i) the virus is acquired by a tick during blood-feeding on an infected host; (ii) the virus is transmitted to a host by a tick that takes its blood-meal after it has molted to the next developmental stage. The period between virus acquisition and virus transmission has been termed the “extrinsic incubation period” during which the tick is not able to transmit the virus (Nuttall, 2009).

The association between a tick species and a transmitted virus is very specific. Indeed, fewer than 10% of the known tick species are suggested to be competent vectors of viruses. These belong to the large tick genera, i.e., soft ticks of the genera *Ornithodoros, Carios*, and *Argas*, and hard ticks of the genera *Ixodes, Haemaphysalis, Hyalomma, Amblyomma, Dermacentor*, and *Rhipicephalus* (Labuda and Nuttall, 2004, 2008; Nuttall, 2014). Most TBV are transmitted either by hard ticks or by soft ticks, but rarely by both (Labuda and Nuttall, 2004). Moreover, some tick species (e.g., *I. ricinus, A. variegatum*) are vectors of a few TBV species, whereas others can transmit several different TBV species (e.g., *Ixodes uriae* is suggested to be the vector of at least 7 TBV) (Labuda and Nuttall, 2008).

During co-evolution, molecular interactions have developed between ticks, TBV and vertebrates, and at their interfaces (Nuttall et al., 1994; Labuda and Nuttall, 2004; Robertson et al., 2009; Mlera et al., 2014; Nuttall, 2014). Natural acquisition of the virus takes place when a tick feeds on an infected vertebrate host or co-feeds with infected ticks on a susceptible uninfected host. The virus enters the midgut, passes through the gut wall, disseminates in the tick body, and reaches the salivary glands (SG) so as to be amplified and transmitted to the next host during subsequent feeding. On its route the virus must cross several barriers in the tick body, such as the midgut infection barrier, midgut release barrier, midgut escape barrier, SG infection barrier, and SG release barrier (Nuttall, 2014).

The best understood TBV transmission cycle is probably that of TBEV (*Flaviviridae*) and its principal vectors, *Ixodes persulcatus* and *I. ricinus*. Several experimental studies have been carried out to explain the TBEV—vector—host interactions. For example, tick infestation of viraemic laboratory animals indicated that most of the tested hard tick species (*Ixodes* spp., *Haemaphysalis* spp., *Dermacentor* spp., *R. appendiculatus*) acquired TBEV from the infected blood meal and maintained the virus transstadially (Rajčáni et al., 1976; Nosek et al., 1984; Kožuch and Nosek, 1985; Alekseev et al., 1988, 1991; Alekseev and Chunikhin, 1990a; Labuda et al., 1993a). TBEV was found to infect tick SG prior to attachment and can be transmitted to a vertebrate host by saliva soon after onset of tick feeding (Řeháček, 1965). Similarly, successful transmission of POWV is likely to occur within 15 min of *I. scapularis* attachment (Ebel and Kramer, 2004), and transmission of Thogoto virus (THOV) within 24 h of attachment of *R. appendiculatus* (Kaufman and Nuttall, 2003). Onset of feeding was found to enhance amplification of TBEV in SG of *I. persulcatus* and *I. ricinus* (Alekseev and Chunikhin, 1990b; Khasnatinov et al., 2009; Slovák et al., 2014a) and of THOV in SG of *R. appendiculatus* (Kaufman and Nuttall, 2003). However, knowledge on the critical stages of TBV survival in their vectors is limited (Nuttall, 2014; Slovák et al., 2014a).

TBV must evade tick innate immune responses in order to persist and replicate in their vectors (Hynes, 2014). In general, tick-borne pathogens have developed different strategies to cope with the tick defense system and high-throughput techniques have already provided insights into both the tick immune responses evoked by bacteria and the bacterial evasion strategies (Smith and Pal, 2014). In contrast, information on molecular mechanisms determining interactions of TBV with ticks is scarce (Hajdušek et al., 2013; Hynes, 2014; Gulia-Nuss et al., 2016). RNA interference (RNAi) appears to be the main antiviral mechanism in ticks that, together with Argonaute (Ago) and endoribonuclease Dicer (Dcr) proteins mediates tick antiviral responses (Schnettler et al., 2014; Gulia-Nuss et al., 2016). However, at present the role of defensin-like peptides displaying *in vitro* virucidal activities against TBV (e.g., longicin or HEdefensin from *Haemaphysalis longicornis*) is unclear in tick antiviral defense (Talactac et al., 2016, 2017).

Tick cell cultures play an important role in many aspects of tick and TBV research (Bell-Sakyi et al., 2012). Since primary tick cell or tissue explant cultures have been introduced, propagation of both arboviruses and non-arthropod-transmitted viruses has been attempted (Řeháček and Kožuch, 1964; Řeháček, 1965; Yunker and Cory, 1967; Cory and Yunker, 1971), and tick cells have been employed to identify and characterize tick genes associated with TBV infection, including those which mediate antiviral activity. For example, by using Langat virus (LGTV)-infected *I. scapularis*-derived cell line, the production of virus-derived small interfering RNAs was revealed (Schnettler et al., 2014). In proteomic studies on *I. scapularis* cells infected with LGTV, 264 differentially expressed tick proteins were identified, out of which the majority were downregulated (Grabowski et al., 2016). The proteins with upregulated expression were associated with cellular metabolic pathways and glutaminolysis. In addition, enzymes that are probably involved in amino acid, carbohydrate, lipid, terpenoid/polykeytide, and vitamin metabolic pathways may also be associated with a decreased replication of LGTV and with a release of infectious virus from *I. scapularis* cells (Grabowski et al., 2017). Analyses of transcriptomes and proteomes of TBEV-infected cell lines derived from *I. ricinus* and *I. scapularis* have identified several molecules that also seem to be involved in the tick innate immune response against flaviviruses and in cell stress responses, such as the heat-shock proteins HSP90, HSP70, and gp96, the complement-associated protein Factor H and trypsin (Weisheit et al., 2015). Furthermore, comparative transcriptome analysis revealed activation of common as well as distinct cellular pathways in *I. ricinus* cells infected with TBEV and LIV and the obligate intracellular bacterium *Anaplasma phagocytophilum*, depending on the infectious agent. Commonly upregulated genes were those that are associated with apoptosis and cellular stress, and genes that affect the tick innate immune responses, whereby only flavivirus infection evoked up- or downregulation of toll genes expression. These data suggest that multiple pathways ensure the control of invading viruses and tick survival (Mansfield et al., 2017).

## Saliva-assisted and non-viraemic transmission

According to Nuttall and Labuda (2008) “saliva-assisted transmission (SAT) is the indirect promotion of arthropod-borne pathogen transmission via the actions of arthropod saliva molecules on the vertebrate host.”

Ticks succeed in feeding by injecting a cocktail of salivary antihaemostatic and immunomodulatory molecules into the feeding pool (Mans and Neitz, 2004; Mans et al., 2008; Kazimírová and Štibrániová, 2013; Wikel, 2014; Chmelar et al., 2016a). The established route of TBV transmission is via an infectious tick bite in which the virus is carried in tick saliva into the feeding lesion in the host skin that is modified by pharmacologically active compounds present in the saliva. Viraemia (infectious virus detectable in circulating blood) in a vertebrate host was considered an important requirement for biological transmission of viruses (World Health Organization Scientific Group, 1985). However, by mimicking natural conditions of transmission using THOV-infected *R. appendiculatus* adults or nymphs that co-fed with uninfected nymphs or larvae on the same naïve guinea pig, a high percentage of the uninfected ticks became infected, although the host animals did not develop detectable viraemia (Jones et al., 1987). Moreover, non-viraemic guinea pigs supported a higher rate of virus transmission between co-feeding ticks than viraemic hamsters. Based on these findings showing that a vertebrate host free of an apparent viremia can play a role in the epidemiology of an arbovirus, a novel mode of arbovirus transmission, “non-viraemic transmission” (NVT), was proposed (Jones et al., 1987). Subsequently, NVT for which the role of tick salivary compounds is anticipated was considered to be indirect evidence of SAT (Nuttall and Labuda, 2008). Furthermore, NVT of THOV even occurred when co-feeding of ticks took place on virus-immune guinea pigs although levels of virus transmission were reduced compared with NVT involving naïve hosts (Jones and Nuttall, 1989a,b). In addition, *R. appendiculatus* was found to be more efficient in mediating NVT than *A. variegatum*, indicating species-specific differences between the vector ticks (Jones et al., 1990a).

Direct evidence of SAT (originally referred to as “saliva-activated transmission”; Jones et al., 1990b) was demonstrated when increased acquisition of THOV was observed in non-infected *R. appendiculatus* nymphs that fed on naïve guinea pigs inoculated with a mixture of the virus and salivary gland extract (SGE) of partially fed *R. appendiculatus* or *A. variegatum* females in comparison to ticks feeding on guinea pigs inoculated only with the virus (Jones et al., 1989). However, SAT was evidenced only when THOV was inoculated along with SGE into tick attachment sites. Viraemia was not detected in the tested animals, suggesting that THOV transmission was enhanced by factors in tick saliva that are likely to mediate NVT. Furthermore, the effect of SAT factor(s) appears to persist in the host skin for several days. Indeed, the proportion of infected *R. appendiculatus* ticks increased when they fed at the skin site where THOV was inoculated 2–3 days after inoculation of SGE (Jones et al., 1992b). SAT factors are likely to be proteins or peptides (Jones et al., 1990b) and enhance virus transmission through immunomodulation of the host rather than by a direct effect on the virus (Jones et al., 1989, 1990b).

Most of the described SAT and NVT events have been associated with hard ticks (Table 2), suggesting that there are differences in the SAT mediators between hard- and soft ticks. Due to their feeding biology (long-lasting blood meal, attachment to hosts in aggregates, and in close proximity), hard ticks appear to be more suitable vectors for NVT than soft ticks (Nuttall and Labuda, 2003). Since the first reports on NVT and SAT, indirect and direct evidence of SAT have been reported for at least 10 different TBV (Table 2).

**Table 2:** Saliva assisted (SAT) and non-viraemic (NVT) transmission of tick-borne viruses.

**Virus**	**Tick species**	**Described effect**	**References**
**SOFT TICKS (ARGASIDAE)**			
***Asfarviridae***			
African Swine Fever virus	*Ornithodoros porcinus*	SAT, host immunomodulation	Bernard et al., 2016
***Flaviridae***			
West Nile virus	*Ornithodoros moubata*	NVT	Lawrie et al., 2004
**HARD TICKS (IXODIDAE)**			
***Orthomyxoviridae***			
Thogoto virus	*Rhipicephalus appendiculatus*	SAT	Jones et al., 1989
		NVT	Jones et al., 1987
	*Amblyomma variegatum*	NVT	Jones et al., 1990a
	*Rhipicephalus evertsi*	NVT	Jones et al., 1992a
	*Amblyomma hebraeum*		
	*Amblyomma cajannense*		
	*Hyalomma dromedarii*		
	*Hyalomma marginatum rufipes*		
	*Amblyomma variegatum*		
	*Boophilus microplus*		
***Flaviviridae***			
TBEV	*Ixodes persulcatus*	NVT	Alekseev and Chunikhin, 1990b
	*Ixodes ricinus*	SAT	Alekseev et al., 1991
			Labuda et al., 1993c
		NVT	Labuda et al., 1993a,b
		SAT, host immunomodulation	Fialová et al., 2010
	*Ixodes scapularis*	SAT, host immunomodulation	Lieskovská et al., 2015
	*Dermacentor reticulatus*	SAT	Labuda et al., 1993c
	*Dermacentor marginatus*		Labuda et al., 1993a,c
	*Rhipicephalus appendiculatus*		
Louping ill virus	*Ixodes ricinus*	NVT	Jones et al., 1997
Powassan virus	*Ixodes scapularis*	SAT	Hermance and Thangamani, 2015
***Bunyavirales***			
CCHFV	*Hyalomma marginatum*	NVT	Gordon et al., 1993
Palma	*Rhipicephalus appendiculatus*	NVT	Labuda et al., 1997a
	*Dermacentor marginatus*		
Bhanja	*Rhipicephalus appendiculatus*	NVT	Labuda et al., 1997a
	*Dermacentor marginatus*		
Heartland virus	*Amblyomma americanum*	NVT	Godsey et al., 2016

Studies based on the THOV model demonstrated NVT for TBEV and *I. persulcatus, I. ricinus, Dermacentor marginatus, D. reticulatus*, and *R. appendiculatus* (Alekseev and Chunikhin, 1990b, 1991; Labuda et al., 1993c). To reproduce natural conditions of TBEV transmission, infected and uninfected *I. ricinus* ticks were allowed to co-feed on naïve wild rodents, the main natural hosts of immature stages. Acquisition of the virus was high in ticks feeding on susceptible *Apodemus* mice (*Apodemus flavicollis, A. agrarius*) that had undetectable or very low levels of viraemia. In contrast, co-feeding transmission was about four-times lower to ticks feeding on tick-resistant bank voles (*Clethrionomys glareolus*) that produced significantly higher viraemia and virus loads in lymph nodes and spleen than *Apodemus* mice (Labuda et al., 1993d).

Similar to THOV, transmission of flaviviruses is mediated by SAT factor(s) (Alekseev et al., 1991; Labuda et al., 1993b). For example, SAT was demonstrated when naïve guinea pigs were inoculated with a mixture of TBEV and SGE derived from partially fed uninfected *I. ricinus, D. reticulatus*, or *R. appendiculatus* females, compared with virus alone, and were infested with uninfected *R. appendiculatus* nymphs. Increased acquisition of the virus was observed in ticks feeding on animals inoculated with the mixture of SGE and virus (Labuda et al., 1993b). Enhancement of POWV transmission by SGE derived from *I. scapularis* has been documented recently; the efficiency of SAT was dependent on the inoculated virus dose (Hermance and Thangamani, 2015). Mice inoculated with a mixture of a high virus dose and SGE as well as with virus alone displayed severe neurological signs of the disease. In contrast, severe clinical signs of the disease were observed in mice inoculated with a low dose of POWV plus SGE, whereas mice inoculated only with a low dose of the virus showed no signs of the disease and displayed low-level viraemia.

SAT has also been documented for bunyaviruses. Transmission of CCHFV from apparently non-viraemic ground-feeding birds to *Hyalomma marginatum rufipes* (Zeller et al., 1994) and between adult *Hyalomma truncatum* co-feeding on naïve rabbits (Gonzalez et al., 1992) was shown. Furthermore, low transmission rates occurred from infected adults of *Hyalomma* spp. to larvae and nymphs that co-fed on non-viraemic guinea pigs (Gordon et al., 1993). NVT appears to be an important amplification mechanism of CCHFV in nature (Bente et al., 2013). Transmission of Palma and Bhanja viruses on non-viraemic laboratory mice was shown by using various donor and recipient tick species (*D. marginatus, D. reticulatus, Rhipicephalus sanguineus, R. appendiculatus*, and *I. ricinus*) (Labuda et al., 1997a). Transmission of the newly described Heartland virus from experimentally infected *A. americanum* nymphs to co-feeding larvae has been documented recently (Godsey et al., 2016).

SAT has rarely been demonstrated in soft ticks. It was reported for the mosquito borne West Nile virus and *Ornithodoros moubata* (Lawrie et al., 2004). In a recent study, modulation of the systemic immune response of domestic pigs and of skin inflammation and cellular responses at the tick bite site by *Ornithodoros porcinus* SGE or feeding was reported. Pigs inoculated with a mixture of AFSV and *O. porcinus* SGE showed greater hyperthermia than pigs inoculated with the virus alone (Bernard et al., 2016).

Although knowledge of the frequency and significance of NVT under natural conditions is still limited, NVT appears to play an important role in the survival of TBV through reducing the pathological impact on the vertebrate host presumably because transmission is more efficient than classical viraemic transmission (Randolph et al., 1996; Labuda and Randolph, 1999; Nuttall and Labuda, 2003; Randolph, 2011).

Several species of wild-living mammals and birds that had not been exposed to TBEV and are known to be natural hosts of *I. ricinus*, were examined for their capacity to support NVT. Infected *I. ricinus* females and uninfected males and nymphs were allowed to co-feed on these animals and were subsequently tested for the presence of TBEV. The examined species differed in their ability to support NVT. While rodents, mainly *Apodemus* mice, were the most efficient amplifying hosts in spite of very low or no detectable viraemia, hedgehogs and pheasants either did not support NVT, or they supported it to a low level. NVT was also observed in bank voles, but their viraemia was higher compared to mice (Labuda et al., 1993d). In order to determine whether virus-immune wild rodents can participate in the transmission of TBEV, yellow necked mice and bank voles were immunized against TBEV and were infested with infected and uninfected *I. ricinus*. In spite of the presence of virus-specific neutralizing antibodies, these animals supported NVT, suggesting that hosts immune to TBEV can participate in the TBEV transmission cycle in nature (Labuda et al., 1997b). Additionally, species-specific differences in the dissemination of TBEV in skin of mice and voles after attachment of infected *I. ricinus* ticks were observed. Indeed, delayed dissemination of the virus from the attachment site of infected ticks to sites where uninfected ticks had fed was confirmed for bank voles but not for mice, partly explaining the difference in the capacity of the two rodent groups to support NVT (Labuda et al., 1996).

NVT plays an important role in the persistence of LIV in nature (Hudson et al., 1995). While red grouse (*Lagopus lagopus scoticus*) are not able to maintain the virus, LIV can persist through NVT in mountain hares (*Lepus timidus*) populations. Mountain hares that did not develop detectable viraemia were shown to support NVT of LIV between co-feeding infected and non-infected *I. ricinus* ticks; the efficiency of NVT was significantly reduced when ticks co-fed on virus-immune hares (Jones et al., 1997).

SAT appears to be correlated with the vector competence of certain tick species for particular viruses (Nuttall and Labuda, 2008). For example, SAT for THOV was demonstrated for *R. appendiculatus* and *A. variegatum* (natural vectors), but not for *I. ricinus* or soft ticks (non-competent vectors) (Jones et al., 1992a). In contrast, SAT for TBEV was observed in natural vectors, *I. persulcatus* and *I. ricinus* (Prostriata), but also in Metastriata species (Alekseev et al., 1991; Labuda et al., 1993b), although *I. persulcatus* and *I. ricinus* appeared to be more efficient donors and recipients of TBEV in NVT than Amblyomminae species (Alekseev and Chunikhin, 1992).

In contrast to the apparent vector specificity of SAT, *D. reticulatus* SGE was found to promote the replication of the insect -borne vesicular stomatitis virus *in vitro* (Hajnická et al., 1998), and the production of the nucleocapsid viral protein (Kocáková et al., 1999; Sláviková et al., 2002) by a yet unexplained mechanism.

### Toward identification of mediators of SAT

The reported cases of SAT do not provide explanations for the molecular mechanisms involved in TBV transmission. During the last two decades, modern molecular-genetic and high-throughput techniques have been applied in the systemic characterization of tick salivary components, enabling elucidation of the underlying molecular mechanisms of exploitation of tick salivary molecules by tick-borne-pathogens (Liu and Bonnet, 2014; Chmelar et al., 2016a,b). Studies on the sialotranscriptome of *I. scapularis* (Valenzuela et al., 2002; Ribeiro et al., 2006) and the *I. scapularis* genome project (Gulia-Nuss et al., 2016) demonstrated the complexity and the redundancy in saliva protein functions within gene families. A wide range of bioactive proteins have been discovered in tick saliva and different expression profiles for a number of genes, depending on the presence or absence of a microorganism, have been described in various tick tissues, including SG (Chmelar et al., 2016a). However, research in this field is more advanced for the tick - tick-borne bacteria interactions than for TBV (e.g., Kazimírová and Štibrániová, 2013; Liu and Bonnet, 2014; de la Fuente et al., 2016b). One of the reasons may be the high pathogenicity of TBV of medical and veterinary importance that require strict conditions for their handling and usage in animal experimentation (e.g., laboratories and animal facilities of biosafety levels 3 and 4). Considering these constraints, usage of less pathogenic models as surrogates for highly pathogenic TBV and research on cell lines offer alternative tools to investigate the processes at the tick—TBV interface.

Until recently, the SG transcript expression profile in response to infection with a TBV has only been described for *I. scapularis* nymphs infected with LGTV (McNally et al., 2012). The study demonstrated that in nymphs feeding for 3 days on naïve mice the number of transcripts associated with metabolism increased in comparison to unfed ticks. A total of 578 transcripts were upregulated and 151 transcripts were downregulated in response to feeding. Differences in expression profiles were revealed also between LGTV-infected and uninfected ticks during the 3 days feeding period. The differently regulated transcripts included putative secreted proteins, lipocalins, Kunitz domain-containing proteins, anti-microbial peptides, and transcripts of unknown function (McNally et al., 2012). A transcript upregulated in LGTV-infected nymphs that belonged to the 5.3 kDa family was previously found to be upregulated in *Borrelia burgdorferi-*infected *I. scapularis* nymphs, suggesting that the protein might play a role in tick immunity or host defense (Ribeiro et al., 2006). However, the specific proteins associated with TBV replication and transmission still need to be identified.

The mechanisms of adaptation of TBV to their vectors and hosts are other important aspects that need to be considered to understand TBV transmission. Specific mutations in the viral envelope protein of TBEV have been found to affect NVT between co-feeding *I. ricinus* for Siberian and European TBEV strains (Khasnatinov et al., 2010). Furthermore, it has recently been demonstrated that the structural genes of the European TBEV strain Hypr may determine high NVT rates of the virus between co-feeding *I. ricinus* ticks, whereas the region of the TBEV genome encoding non-structural proteins determines cytotoxicity in cultured mammalian cells (Khasnatinov et al., 2016).

## Immunomodulation of host immune cells at the tick attachment site—A prerequisite for TBV transmission

The redundant host defense mechanisms in the skin pose a significant threat to successful tick feeding; however, tick saliva contains an array of pharmacologically active compounds that are vital to overcoming haemostasis, wound healing, and innate and adaptive immune responses of the host. Among the repertoire of bioactive tick salivary molecules are inhibitors of the pain and itch response, anticoagulants, antiplatelet components, vasodilators, and immunomodulators, all of which have been extensively highlighted in several comprehensive reviews (Ribeiro and Francischetti, 2003; Ribeiro et al., 2006; Francischetti et al., 2009; Kazimírová and Štibrániová, 2013; Wikel, 2013). As a tick feeds, salivation is not a continuous process (Kaufman, 1989). Expression of a plethora of tick salivary proteins was found to be differentially up- or downregulated during blood feeding, and differences in saliva composition exist across and within tick genera (McSwain et al., 1982; Ribeiro et al., 2006; Alarcon-Chaidez et al., 2007; Vančová et al., 2007, 2010a; Mans et al., 2008; Peterková et al., 2008; Francischetti et al., 2009; Kazimírová and Štibrániová, 2013; Wikel, 2013). Thus, the composition of tick saliva is dynamic and complex so that it may overcome the many redundancies inherent to the host defenses (Kazimírová and Štibrániová, 2013; Kotál et al., 2015).

**Figure 1:**
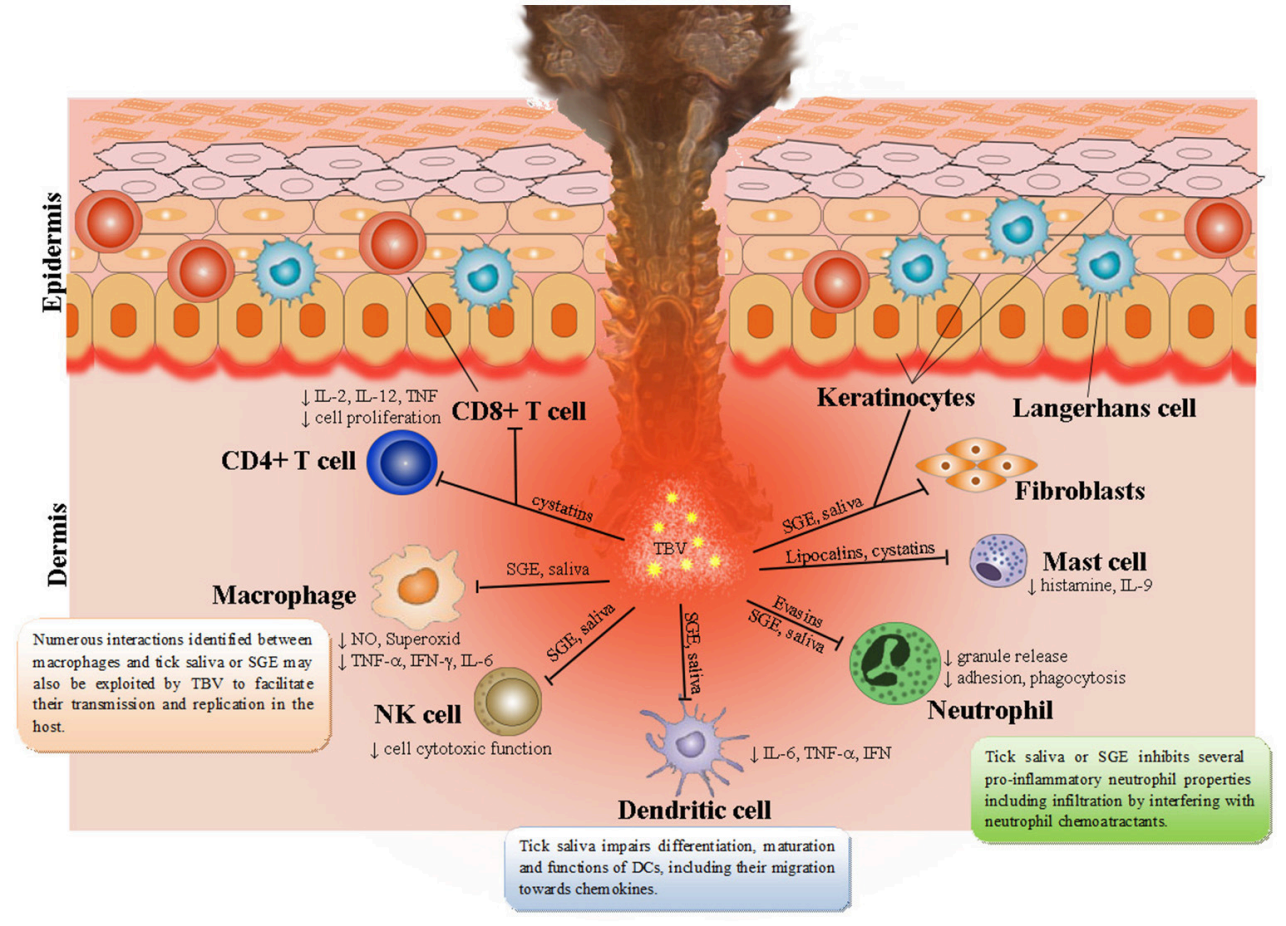
Tick saliva contains a broad spectrum of pharmacologically-active molecules affecting various immune cell populations. Skin is the key interface for tick-virus-host interactions. Resident skin cells—keratinocytes, Langerhans cells (epidermal dendritic cells), dendritic cells, T cells, macrophages, fibroblasts, and endothelial cells are immediately activated after first contact with tick saliva, hypostome, and TBVs. By producing and releasing a wide range of pro-inflammatory chemokines and cytokines they recruit other immune cells, such as neutrophils, T cells, and B cells into the tick attachment site. Tick saliva modulates immune responses to facilitate feeding and consequently facilitate transmission of TBVs, which target and replicate in different skin cells including keratinocytes, dermal macrophages, Langerhans' DCs, and neutrophils.

Ticks are able to modulate cutaneous as well as systemic immune defenses of their hosts that involve keratinocytes, natural killer (NK) cells, dendritic cells (DCs), T cell subpopulations (Th1, Th2, Th17, T regulatory cells), B cells, neutrophils, mast cells, basophils, endothelial cells, cytokines, chemokines, complement, and extracellular matrix (Kazimírová and Štibrániová, 2013; Štibrániová et al., 2013; Wikel, 2013; Heinze et al., 2014) (Figure 1). The general pattern of tick infestation- or tick saliva-induced immunomodulation consists of downregulation of Th1 cytokines and upregulation of Th2 cytokines leading to suppression of host antibody responses. The dynamic balance between host immunity and tick immunomodulation has been found to affect both tick feeding and pathogen transmission (Bowman et al., 1997; Ramamoorthi et al., 2005; Brossard and Wikel, 2008; Nuttall and Labuda, 2008; Wikel, 2013).

Skin is the first host organ that TBV and tick saliva encounter during the tick feeding process. In addition to serving as the host's primary line of defense from the outside environment (Nestle et al., 2009), skin is also the interface for tick-virus-host interactions (Nuttall and Labuda, 2004; Wikel, 2013). Thus, cutaneous immune cells play a crucial role in the initial response of the host to tick feeding and invading pathogenic microorganisms, including viruses (Labuda et al., 1996; Frischknecht, 2007; Wikel, 2013, 2014; Hermance and Thangamani, 2014; Bernard et al., 2015; Hermance et al., 2016). Penetration through the skin brings tick mouthparts into contact with keratinocytes, which possess receptors of innate immune responses, antimicrobial peptides, and pro-inflammatory cytokines (Merad et al., 2008; Martinon et al., 2009; Nestle et al., 2009). TBV delivered into the skin also encounter different cell types, including rich DC networks and neutrophils, which are involved in pathogen elimination during the early stages of infection (Labuda et al., 1996; Wu et al., 2000; Robertson et al., 2009). TBV were found to replicate at the tick bite site within keratinocytes, dermal macrophages, Langerhans' DCs, and neutrophils (Wikel et al., 1994; Labuda et al., 1996; Wu et al., 2000; Ho et al., 2001; Libraty et al., 2001; Marovich et al., 2001).

### Modulation of dendritic cell functions

Recognition of TBV by immature DCs occurs via pattern recognition receptor (PRRs) systems such as Toll-like receptors (TLRs) located at the cell surface and within endosomes, or the retinoic acid-inducible gene I (*Rig-1*)-like helicases (RLHs) detecting nucleic acids within the cytosol (Kochs et al., 2010). DCs are known to take up viral antigens which results in DC activation and their migration to local lymphoid tissues. As DCs are the key players in the induction of protective immunity to viral infection, tick salivary molecules that modulate DC functions are probably exploited by viruses to circumvent host immune responses. During the early phase of infection, a virus replicates within the dermis and subsequently in the skin draining lymph nodes. Activation of DCs confers their ability to activate naïve T cells into T helper type 1 (Th1), Th2, and cytotoxic T lymphocyte (CTL) effector cells. This interaction activates signaling pathways that lead to increased expression of major histocompatibility complex (MHC) class II molecules (required for antigen presentation), T cell co-stimulatory molecules (i.e., CD80 and CD86), and proinflammatory cytokines, such as type I interferon (IFN), interleukin (IL) 6, and IL12, which drive anti-viral Th1 responses (Johnston et al., 2000; Masson et al., 2008).

Tumor necrosis factor (TNF)-alpha is a powerful cytokine secreted by several cell types after viral infection. Together with IL1, TNF-alpha is known to promote DC migration from the skin into regional lymph nodes. TBEV infection was found to induce the release of TNF-alpha and IL6 by DCs, but undetectable levels of IL10 and IL12p70 were measured in DC cultures infected with the virulent Hypr strain in contrast to DCs infected with the less virulent Neudoerfl strain (Fialová et al., 2010). Treatment with *I. ricinus* saliva was found to prolong the survival of TBEV-infected DCs, and suppress the levels of TNF-alpha and IL6, but at the same time, bystander DCs kept the immature phenotype as assessed by low expression of B7-2 and MHC class II molecules (Fialová et al., 2010). Similar results were previously reported for uninfected DCs treated with *I. ricinus* saliva (Sá-Nunes et al., 2007; Hovius et al., 2008). It has been proposed that *I. ricinus* saliva impairs maturation of murine DCs through affecting TLR3, TLR7, TLR9, or CD40 ligation, and reduced TBEV-mediated DCs apoptosis (Skallová et al., 2008). The findings may suggest that in the presence of tick saliva, DCs keep a less mature phenotype and therefore remain permissive for TBEV. On the other hand, tick saliva does not affect virus-induced upregulation of MHC class II and B7-2 molecules.

Differentiation, maturation, and functions of DCs were found to be impaired by *R. sanguineus* saliva (Cavassani et al., 2005) and prostaglandin (PG)E2 from *I. scapularis* saliva (Sá-Nunes et al., 2007). Co-incubation of DCs with *R. sanguineus* saliva promoted attenuation of antigen-specific T cells cytokine production stimulated by DCs (Oliveira et al., 2008). Moreover, saliva of *R. sanguineus* impaired the maturation of DCs stimulated with lipopolysaccharide (LPS), a TLR-4 ligand, by inhibition of the activation of the ERK 1/2 and p38 MAP kinases, leading to increased production of IL10 and reduced synthesis of IL12p70 and TNF-alpha (Oliveira et al., 2010). The above observations suggest that in the presence of tick saliva infected DCs may stay in the skin for a prolonged time and serve as a further source of the virus, which may, together with the saliva-induced impairment of DC migration, enhance NVT.

Tick saliva was also found to inhibit the chemotactic functions of chemokines and selectively impair chemotaxis of immature DCs by downregulating cell-surface receptors. Saliva of *R. sanguineus* inhibits immature DC migration in response to CCL3 (migration via receptors CCR1 or CCR5), to CCL4 (MIP-1 alpha) (via CCR1), and to CCL5 (RANTES) (migration via CCR1, CCR3, CCR5) (Oliveira et al., 2008). Evasin-1 (derived from *R. sanguineus*) is also able to bind to human CCL3 and mouse CCL3 (Dias et al., 2009). Two salivary cystatins (cysteine protease inhibitors) derived from *I. scapularis* have been shown to inhibit cathepsins L and S, impair inflammation, and suppress DC maturation (Kotsyfakis et al., 2006, 2008; Sá-Nunes et al., 2009). As a result, saliva of ticks can impair early migration of DCs from inflamed skin.

Reduction in the number of DCs was observed around the attachment sites of *D. andersoni* ticks, suggesting that migration of Langerhans cells to lymph nodes occurs after contact with tick salivary components and T cell responses. *In vitro* treatment of DCs from the lymph nodes of tick-bite sensitized tick-resistant guinea pigs with tick saliva induced T cell proliferation (Nithiuthai and Allen, 1985), and co-incubation of DCs with tick saliva lead to attenuation of antigen-specific T cells cytokine production stimulated by DCs (Oliveira et al., 2008). In addition, density and recruitment of Langerhans cells were inhibited by inoculation of SGE or feeding of *O. porcinus* in the skin of domestic pigs infected with ASFV, demonstrating immunomodulatory capacities also for soft tick saliva (Bernard et al., 2016).

A novel mechanism of immunomodulation, potentially facilitating pathogen transmission, has been discovered by Preston et al. (2013). Japanin, a new member of saliva lipocalins from *R. appendiculatus* ticks, was found to specifically reprogram DC responses to a wide variety of *in vitro* stimuli. Japanin was found to alter the expression of co-stimulatory and co-inhibitory transmembrane molecules, modulate secretion of pro-inflammatory, anti-inflammatory, and T cell polarizing cytokines, and also inhibit the differentiation of DCs from monocytes. Based on these findings it was suggested that the failure of DCs to mature in response to viral or tick immunomodulators has important implications for induction of effective antiviral T cell mediated immunity, i.e., it may lead to an aberrant anti-viral immune response and ineffective virus clearance.

### Modulation of interferon signaling

Although DCs represent an early target of TBV infection, they are major producers of IFN. It has been shown that both early DC and IFN responses are modulated by viruses (Best et al., 2005), but also by tick salivary immunomodulatory compounds. Generally, following virus infection, the host cells deploy the rapid response to limit virus replication in both infected cells and in neighboring cells. Indeed, the IFN-dependent innate immune response is essential for protection against flavivirus infections, whereby type I IFN (including multiple IFN-alpha molecules and IFN-beta) have a central role (Akira et al., 2006; Kawai and Akira, 2006). Although type I IFN signaling is recognized as an important component of antiviral innate immunity, previous studies indicate that its role during vector-borne flavivirus infection is complex and varies, depending on the virus species. Type I and II IFN were found to inhibit flavivirus infection in cell culture and in animals. Type I IFN (alpha or beta) blocks flavivirus infection by preventing translation and replication of infectious viral RNA, which occurs at least partially through an RNAse L, Mx1, and protein kinase (PKR)-independent mechanism. Mx1 and MxA proteins have been determined as the innate resistance factors in mammalian cells against tick-borne orthomyxoviruses (THOV and Batken virus) (Halle et al., 1995; Frese et al., 1997) and bunyaviruses (CCHFV and Dugbe virus) (Andersson et al., 2004; Bridgen et al., 2004). Possible manipulation of IFN signaling by tick SGE was indicated by Dessens and Nuttall (1998) who demonstrated THOV transmission to uninfected ticks feeding on Mx1 A2G mice (a strain resistant to infection) following needle- or tick-borne virus challenge, probably thanks to *Mx1* gene manipulation after injection of virus mixed with tick SGE.

LGTV, a member of the TBEV complex, is sensitive to the antiviral effects of IFN. LGTV-mediated inhibition of JAK-STAT signaling as well as interactions between NS5 and IFNAR2, were demonstrated in infected human monocyte-derived DCs. Non-structural NS5 protein blocked STAT1 phosphorylation in response to either IFN-alpha or IFN-gamma. An association was observed between NS5 and both the IFN-alpha/beta receptor subunit, IFNAR2 (IFNAR2-2 or IFNAR2c), and the IFN-gamma receptor subunit, IFNGR1 (Best et al., 2005). However, arboviruses are generally not recognized as strong inducers of IFN-alpha/beta, with one exception, vesicular stomatitis virus, an insect-borne rhabdovirus. Using this virus, Hajnická et al. (1998, 2000) were the first who provided evidence that SGE from partially fed adult *R. appendiculatus* or *D. reticulatus* increased viral yields by 100- to 1,000- fold in mouse cell cultures. The effect appeared to be due to inhibition of the antiviral effect of IFN by SG factors, possibly acting through the IFN-alpha/beta receptor rather than directly affecting IFN.

The recently observed enhanced replication of TBEV in bone marrow DCs in the presence of *I. scapularis* sialostatin L2 is probably a consequence of impaired IFN-beta signaling (Lieskovská et al., 2015). Both sialostatin L and sialostatin L2 decreased STAT-1 and STAT-2 phosphorylation, and inhibited IFN stimulated genes, *Irf-7* and *Ip-10* in LPS-stimulated DCs. The inhibitory effect of tick cystatin on IFN responses in host DCs appears to be a novel mechanism by which tick saliva assists in the transmission of TBV.

Immune IFN, known as type II IFN or IFN-gamma, is secreted mostly by activated NK cells and macrophages during the early stages of infection (Malmgaard, 2004; Darwich et al., 2009). During later stages of infection, IFN-gamma is produced by activated T lymphocytes (Boehm et al., 1997) in answer to receptor-mediated stimulation (through T cell receptors or NK cell receptors) or in response to early produced cytokines, such as IL12, IL18, and IFN-alpha/beta (Darwich et al., 2009). Type II IFN (IFN-gamma) inhibits flavivirus replication via the generation of pro-inflammatory and antiviral molecules including nitric oxide (NO). Antiviral activity is not the primary biological function of IFN-gamma. However, through stimulation of the activation of macrophages and increasing the expression of MHC for more effective antigen presentation, IFN-gamma can enhance cell-mediated immune responses that are critical for the development of immunity against intracellular pathogens. It was shown that SGE of *I. ricinus* reduced polyinosinic-polycytidylic acid (poly IC)-induced production of IFN-alpha, IFN-beta, and IFN-gamma (Kopecký and Kuthejlová, 1998) and SGE of female *D. reticulatus* inhibited antiviral effects of IFN-alpha and IFN-beta produced by mouse fibroblasts (Hajnická et al., 2000). SGE from 5-days fed *D. reticulatus* and *I. ricinus* females were shown to inhibit ConA stimulated IFN-gamma production by mouse splenocytes (Vancová, unpublished).

A variety of viruses and different TLR agonists can stimulate Type III IFN (IFN-lambda) gene expression in a similar manner as the expression of type I IFN genes that is induced by transcriptional mechanisms involving IRF's and NF-κB (Onoguchi et al., 2007; Osterlund et al., 2007). Among skin cell populations, keratinocytes and melanocytes, but not fibroblasts, endothelial cells or subcutaneous adipocytes, are targets of IFN-lambda (Witte et al., 2009). Keratinocytes are cells that produce and respond to type III IFN (Odendall et al., 2014). IFN-lambda probably acts primarily as a protection of mucosal entities, e.g., in the lung, skin, or digestive tract (Hermant and Michiels, 2014). According to results provided by Lim et al. (2011), Limon-Flores et al. (2005) and Surasombatpattana et al. (2011), keratinocytes were proposed as key players of early arboviral infection capable of producing high levels of infectious virus in the skin favoring viral dissemination to the entire body.

### Modulation of macrophage functions

Macrophages are potential targets for TBV. Infection of macrophages with flaviviruses leads to production of NO, which inhibits virus replication (Plekhova et al., 2008). However, the exact roles of NO produced by macrophages in the context of TBV infection remain to be elucidated. It has been demonstrated that mononuclear/macrophage lineages are important sources of local TBEV replication before viraemia occurs (Dörrbecker et al., 2010). Thus, modulation of macrophage functions by tick saliva may also be exploited by TBV to facilitate their transmission and replication in the host. Resident macrophages in the skin act as antigen-presenting cells that elicit a potent proliferative response during secondary tick infestation. Macrophages recruit in increased numbers to the site of injury in response to inflammatory and immune stimulation, and produce cytokines and chemokines that attract inflammatory cells to the tick bite site. The tick macrophage migration inhibitor factor, MIF, identified in SG of *A. americanum*, might impair macrophage functions during virus infection (Jaworski et al., 2001). Moreover, tick saliva was shown to decrease the oxidative activity of mouse macrophages (Kuthejlová et al., 2001).

### Modulation of neutrophil functions

In addition to DCs and macrophages, neutrophils are recruited to the site of TBV infection (Dörrbecker et al., 2010; Hermance et al., 2016). Neutrophils probably play a role in complementing the cytokine and chemokine responses soon after TBV infection. They may also be involved in the peripheral spread of TBV. However, at the present stage of knowledge we can only speculate about the exploitation of tick saliva neutrophil inhibitors by TBV.

At the tick attachment site, neutrophils are activated by thrombin from the blood-coagulation cascade, by platelet-activating factor, by releasing of proteases modulating platelet function, such as cathepsin G, and/or enzymes that act on tissue matrix, like elastase. Neutrophils are the most abundant cells in the acute inflammatory infiltrate induced by primary tick infestation, but not during subsequent infestations, at least not by all tick species and not in all tick—host associations (Brown, 1982; Brown et al., 1983, 1984; Gill and Walker, 1985). Ticks were found to generate a neutrophil chemotactic factor in their saliva by cleavage of C5 (Berenberg et al., 1972).

Neutrophil infiltration and activation is orchestrated by chemokines such as CCL3, CXCL8/KC. It has been demonstrated that SGE of different hard tick species effectively bind and block in action a broad spectrum of pro-inflammatory cytokines and chemokines. A number of tick species were shown to possess anti CXCL8 activity mediated by one or more molecules (Hajnická et al., 2005, 2001; Vancová et al., 2010b). Inhibition of CXCL8-coordinated neutrophil migration due to inhibition of CXCL8-binding to the cell receptors was demonstrated for *D. reticulatus* SGE (Kocáková et al., 2003). Evasin-1 and Evasin-3 were identified as potent inhibitors of CCL3 and/or CXCL8-induced recruitment of human and murine neutrophils (Déruaz et al., 2008).

### Wound healing

Many tick saliva molecules are involved in modulation of epithelial wound healing and vasculature repair, including cytoskeletal elements (Maxwell et al., 2005; Heinze et al., 2012). Wound-healing events, initiated by haemostasis, are orchestrated by cytokines, chemokines, and growth factors (Behm et al., 2011). Platelets, macrophages, fibroblasts, and keratinocytes release growth factors that initiate a downstream response to promote wound healing. Hypoxic milieu in the wound results in reactive oxygen species (ROS) production and cytokine release. Fibroblast growth factor 7 (FGF-7)-activated peroxiredoxin-6 and Nrf-2 transcription factor protect cells, especially keratinocytes and macrophages, from ROS-induced damage. FGF-2, 7, and 10 are essential in the proliferative phase of wound healing, neoangiogenesis, and re-epithelization. IL1 and IL6 are important in inflammation, angiogenesis, and keratinocyte migration; they affect tissue remodeling by regulation of matrix metalloproteinases (MMPs) and tissue inhibitors of MMPs. IL1 molecules are among the first signaling molecules released by keratinocytes and leukocytes in response to disruption of the epidermal barrier. IL1, IL6, and TNF-alpha-activated hepatocyte growth factor (HGF) production in fibroblasts increase tissue granulation, neoangiogenesis, and re-epithelization (Toyoda et al., 2001). IL6, produced by fibroblasts, macrophages, endothelial cells, and keratinocytes play important roles in all steps of wound healing. Through downstream mechanisms, IL6 induces neutrophil and macrophages infiltration, collagen deposition, angiogenesis, and epidermal cell proliferation. Re-epithelization in wound healing is enhanced by the epidermal growth factor family (EGF). Activated macrophages release platelet-derived growth factor (PDGF) and TGF-beta1 which attract leukocytes, fibroblasts and smooth muscle cells to the wound site in the skin. Infiltration of leukocytes in inflammatory tissues is mediated by the intracellular adhesion molecule-1 (ICAM-1) and vascular cell adhesion molecule -1 (VCAM- 1). Significant down-regulation of ICAM-1 expression by SGE of *D. andersoni* ticks, and significant reduction of VCAM-1 expression by *I. scapularis* SGE were described (Maxwell et al., 2005). Leukocytes and monocytes actively produce growth factors that prepare the wound for the proliferative phase, when fibroblasts and endothelial cells are recruited. TGF-beta1 that controls signals of fibroblast functions is produced by activated platelets, macrophages and T lymphocytes and affects extracellular matrix deposition, and increases collagen, proteoglycans and fibronectin gene transcription. Furthermore, TGF-beta1 stimulates the tissue metalloprotease inhibitor, and other cytokines (interleukins, fibroblast growth factor FGF, TNF-beta3). TGF-beta1 binding activity, as well as other growth factor binding activities (PDGF, HGF, FGF2) have been detected in saliva of *D. reticulatus, R. appendiculatus, I. ricinus, I. scapularis, A. variegatum*, and *H. excavatum* ticks (Hajnická et al., 2011; Slovák et al., 2014b). Kramer et al. (2011) identified a stimulating effect of *D. variabilis* saliva on basal-and PDGF-stimulated migration of macrophage derived cell line IC-21. In the inflammatory phase of wound healing and angiogenesis, macrophages may transform to produce proliferative mediators in response to IL4 released by mast cells and leukocytes. This switch stimulates collagen synthesis and fibroblast proliferation. Feeding of *D. andersoni* was found to regulate cell signaling, phagocytosis and gene expression, and skewed the immune response toward a Th2 profile, which is characterized by production of anti-inflammatory cytokines IL4 and IL10 (Kramer et al., 2011). Interleukins and TGF-beta are crucial regulators of MMPs that are important for matrix remodeling and angiogenesis (Boniface et al., 2005; Lamar et al., 2008). Chemokines produced by keratinocytes (CXCL11) and by neovascular endothelium (CXCL10) (IP10) are crucial in signaling of the regenerative wound healing phase. Both interact with CXCR3; activation of CXCR3 signaling converts fibroblast from migratory to a contractile state following maturation of collagen fibers (Satish et al., 2003). Modulation of the wound healing processes by tick bioactive compounds may also be exploited by TBVs.

## Early host cutaneous changes at the tick attachment site

Several studies have used cutaneous feeding site lesions from uninfected ticks to examine the tick-induced changes in cutaneous gene expression and histopathology during the early stages of uninfected tick feeding. Early transcriptional and histopathological changes at the feeding site of uninfected *I. scapularis* nymphs are initially characterized by modulation of host responses in resident cells, followed by progression to a neutrophil-dominated immune response (Heinze et al., 2012). When the cutaneous immune responses and histopathology were analyzed during uninfected *D. andersoni* nymph feeding, chemotaxis of neutrophils and monocytes into the feeding site and keratin-based wound healing responses were prominent (Heinze et al., 2014). During the early phase of primary infestation by *D. andersoni*, significant upregulation of the genes for chemokines (*Ccr1, Ccl2, Ccl6, Ccl7, Ccl12, Cxcl1, Cxcl2*, and *Cxcl4/Pfx4*), cytokines (*Il1b*) and anti-microbial molecules, and downregulation of genes related to DNA repair, transcription, chromatin remodeling, transcription factor binding, RNA splicing, and mRNA metabolism was demonstrated (Heinze et al., 2014). In addition, upregulation was found for the genes for *Nfkbia* and *Tsc22d3* which inhibit NF-κB and AP-1 pro-inflammatory pathways (Heinze et al., 2014). NF-κB and NFAT were previously identified as two of the most important factors coordinating mechanisms of viral evasion by regulation of pro-inflammatory molecules and cytokines which evoke inflammatory responses and recruitment of immune cells (Kopp and Ghosh, 1995). Moreover, during early and late primary *D. andersoni* infestation, murine host genes (*Cyr61, SMAD5, TNFrsf 12, Junb, Epgnc*) that may be related to TNF-alpha, AP-1, and growth factor responses at the tick bite site, were upregulated while genes encoding cytoskeletal elements (*collagen type 1 gene, laminin beta2*), signaling molecules, growth factor receptor (*Pdgfrb*), or growth factor (*Tgfb3*) were downregulated (Heinze et al., 2014). The experiments with cutaneous feeding site lesions from uninfected ticks set the stage for studying the role of localized skin infection and the cutaneous immune response during virus-infected tick feeding.

### Cutaneous immune response to tick-borne flavivirus-infected tick feeding

Due to the fact that flaviviruses can be transmitted within 15 min of tick attachment (Ebel and Kramer, 2004), attention has been focused on the early stages of tick feeding and TBV transmission. It has long been suspected that localized immunomodulation induced by tick saliva and the cellular infiltrates recruited to the tick feeding site can facilitate TBV replication and transmission, however, there are a limited number of studies that have directly investigated this phenomenon *in vivo*. Prior to gene expression analysis conducted at the POWV-infected *I. scapularis* tick feeding site, no study had used an *in vivo* model to characterize the host's cutaneous immune response during the early stages of TBV transmission. Comparative gene expression analysis between POWV-infected and uninfected *I. scapularis* tick feeding sites was performed at 3 and 6 h after tick attachment (hours post-infection, hpi). After 3 h of POWV-infected tick feeding, cutaneous gene expression analysis revealed a complex proinflammatory environment, which included significant upregulation of proinflammatory cytokines related to neutrophil and phagocyte recruitment, migration, and accumulation (Hermance and Thangamani, 2014). In contrast to the 3 hpi time point, the majority of significantly modulated genes at 6 hpi were down-regulated, including several proinflammatory cytokines associated with the inflammatory response reaction, suggesting decreased recruitment of granulocytes at the later time point (Hermance and Thangamani, 2014). These data suggest that POWV-infected tick feeding recruits immune cells earlier than uninfected tick feeding.

The murine cutaneous immune response during the early stages of POWV-infected tick feeding was further examined by immunophenotyping infiltrating immune cells and identifying cell targets of POWV infection at the *I. scapularis* feeding site (time points ≤24 hpi). The most distinct histopathological difference between the POWV-infected vs. uninfected tick feeding sites was observed at 3 hpi, when higher levels of cellular infiltrates (mostly neutrophils and some mononuclear cells) were detected at the POWV-infected tick feeding sites compared to the uninfected feeding sites (Hermance et al., 2016). These histopathological findings correlate with gene expression analysis, and together the results demonstrate that neutrophil and mononuclear cell infiltrates are recruited earlier to the feeding site of a POWV-infected tick vs. an uninfected tick (Hermance et al., 2016). Furthermore, POWV antigen was detected in macrophages and fibroblasts located at the tick feeding site, which suggests that these cells are early targets of infection (Hermance et al., 2016). No prior studies used an *in vivo* tick feeding model to report on immune cell targets of infection at the skin interface and the cutaneous immune response during the early hours of tick-borne virus transmission. These findings highlight the complexity of the initial interactions between the host immune response and early tick-mediated immunomodulation, all of which initially occur at the skin interface.

### Localized skin infection during the early transmission of tick-borne flavivirus

The tick attachment and feeding site plays a crucial role in establishing a focus of viral replication during early virus transmission and establishment in the host. This phenomenon was first demonstrated with TBEV where conditions mimicking natural TBEV transmission were incorporated into the experimental design by allowing infected and uninfected *I. ricinus* ticks to co-feed on the same murine host (Labuda et al., 1996). These experiments demonstrated that TBEV is preferentially recruited to tick-infested skin sites compared to uninfested skin sites, and co-feeding TBEV transmission is dependent on localized skin infection at tick feeding sites as opposed to an overt viremia (Labuda et al., 1996). Furthermore, *ex vivo* data from this study suggests that immune cells infiltrating the skin site during tick feeding, and subsequently migrating from such sites, serve as vehicles for TBEV transmission between co-feeding ticks, a process independent of a systemic viremia (Labuda et al., 1996).

Certain immune cells are likely involved in virus dissemination as they emigrate from the skin site of tick feeding. Langerhans cells are the main DC subpopulation in the epidermis, and their major function is to capture antigens in the epidermis and migrate to skin-draining lymphoid tissues where the appropriate immune response is initiated. Langerhans cell migration to draining lymph nodes has been demonstrated in response to cutaneous infections with arboviruses such as West Nile virus and Semliki Forest virus (Johnston et al., 2000). Consequently, in the *ex vivo* experiments conducted by Labuda et al. (1996), the presence of TBE viral antigen in emigrating Langerhans cells suggests that these cells serve as vehicles for TBEV transportation to the lymphatic system, a phenomenon that contributes to overall viral dissemination. The importance of virus-infected cells at the tick feeding site and their contribution to initial viral replication and dissemination was further supported by *in vitro* experiments where *I. ricinus* tick saliva was shown to modulate TBEV infection of dendritic cells. Specifically, when DCs were cultured with TBEV in the presence of *I. ricinus* saliva, the infection rate of the cells was enhanced and there was a decrease in virus-induced TNF- alpha and IL6 production (Fialová et al., 2010). Together these studies illustrate the important role of localized skin infection during the early stages of tick-borne flavivirus transmission.

## Vaccines

Globally, the epidemiological impact of TBV infections is small in the context of infectious diseases. This is one reason why there are comparatively few vaccines available for controlling tick-borne viral diseases. There is also the challenge of developing vaccines effective against topological variants of the diverse strains of given viral species. One approach to overcoming this challenge is to develop vaccines that target important tick vector species in such a way that they interfere with the transmission of all tick-borne viruses. Here we review briefly the current state of anti-tick vaccines, and consider future prospects.

An anti-tick vaccine has been marketed since 1994 under the trade name TickGARD; a Cuban version is marketed as Gavac (Willadsen, 2004). The vaccines derive from Bm86/Bm95, midgut antigens of the cattle tick, *R*. (*B*.) *microplus*. They work by eliciting antibodies in immunized animals. When taken up in the bloodmeal of feeding ticks, the antibodies bind to the antigen resulting in damage to the midgut. The consequent impact on feeding success and reproductive output causes a gradual reduction in tick numbers and tick-borne infections (de la Fuente et al., 2007). Despite many attempts to develop a more efficacious vaccine, none has been commercialized.

Development of anti-tick vaccines is driven by the need to control tick infestations of livestock and the diseases caused by tick-borne pathogens, and the increasing resistance of cattle tick populations to commonly used acaricides (Schetters et al., 2016). Considerable effort is directed against the cattle tick [*R*. (*B*.) *microplus*] although other species (e.g., *H. longicornis, H. anatolicum, I. ricinus*) are being investigated. Most strategies favor the “hidden” or “concealed” antigen approach, as illustrated by the Bm86/Bm95-derived anti-tick vaccines. These are antigens not normally exposed to the host immune system during blood feeding. The most promising candidates include subolesin, ferritins, and aquaporins. Tick subolesin is functionally related to mammalian akirin-2, a downstream effector of the Toll-like receptor required in the innate immune response. A combination of subolesin and Bm86 has been patented as a more effective formulation for controlling cattle tick infestations (Schetters and Jansen, 2014). Ferritins help ticks cope with the potentially toxic heme from the bloodmeal. Ferritin 2 (Fer2) is a target for vaccine development because it is expressed in the midgut where it mediates transportation of nonheme iron to peripheral tissues, and it is not found in mammals (Hajdusek et al., 2009). Vaccination of cattle with recombinant cattle tick Fer2 elicited protection at least comparable to the Bm86 control antigen (Hajdusek et al., 2010). Aquaporins are integral membrane proteins that serve as channels for the transfer of water across cell membranes. Ticks use aquaporins to remove water from the bloodmeal, a critical process for osmoregulation (Campbell et al., 2010). A recombinant cattle tick aquaporin provided >65% efficacy in two cattle pen trials of a vaccine formulation (Guerrero et al., 2014). However, aquaporins are ubiquitous hence extensive safety testing is needed before they can be licensed in vaccines. Moreover, induced immunity to concealed antigens takes time to act and is usually short-lived.

The alternative “exposed” antigen approach targets proteins or peptides secreted by ticks when they feed and which therefore are exposed to the immune response of the tick-infested host. Several such exposed antigens have been evaluated as recombinant protein anti-tick vaccines with disappointing results (Nuttall et al., 2006; Olds et al., 2016). The concern about this approach is the remarkable diversity (so-called “redundancy”) of tick saliva proteins and the likelihood that immune selection pressure on the targeted secreted antigen results in ticks overcoming the vaccine effect.

A third strategy for anti-tick vaccine development is the “dual action” approach involving a secreted (“exposed”) antigen that cross-reacts with a midgut (“concealed”) antigen (Nuttall et al., 2006). This strategy benefits from the boosting effect of a conserved secreted antigen (the feeding tick elicits an anamnestic response in a vaccinated host), hence inducing long-lasting immunity, while damaging the tick midgut. At least two antigens have been shown to have this dual action: 64TRP cement protein antigen from *R. appendiculatus* and OmC2 cysteine peptidase inhibitor from *O. moubata* (Trimnell et al., 2002; Salát et al., 2010). Trials in cattle of a 64TRP-based vaccine were reported by Merial to show promising results although the data were not published and vaccine development ceased.

More recently, new technologies have made tractable approaches based on a deeper understanding of complex tick-pathogen-host interactions (de la Fuente et al., 2016a; Kuleš et al., 2016). For example, SILK (a salivary gland-expressed flagelliform protein of unknown function) and TROSPA (tick receptor for OspA localized in the gut) facilitate transmission of cattle tick-borne pathogens, *Anaplasma marginale* and *Babesia bigemina*, respectively. While vaccination with SILK reduced tick infestations, oviposition, and levels of *A. marginale* and *B. bigemina* DNA, vaccination with TROSPA did not have a significant effect on any of the tick parameters analyzed and *B. bigemina* (but not *A. marginale*) DNA levels were reduced (Merino et al., 2013). Neither SILK nor TROSPA were significantly more effective than subolesin in reducing tick infestation/productivity or pathogen DNA levels although subolesin is not a recognized facilitator of pathogen transmission and infection. These results illustrate the need for a better understanding of the interface between feeding tick and immunized host/bloodmeal (at the skin site of attachment and within the tick midgut) and how these tick-host interactions affect tick-borne pathogens.

The prospects of developing a single anti-virus vaccine against all TBVs are unrealistic at this point in time as no common target has yet been identified against which vaccines can be developed. However, the idea of a single anti-tick vaccine that provides universal protection against TBV infections is not quite so far-fetched given that TBVs have a common target: they are reliant on a tick vector to survive. Thus, if a tick antigen is found that is common to tick vector species, and immunization with the antigen induces a host response that interferes with virus transmission, the possibility of developing a universal TBV vaccine becomes real. The “wish list” for an ideal universal TBV vaccine looks something like this:

1. Effective against a wide variety of tick species and against different tick developmental stages;
2. Inhibits or suppresses transmission of all viruses vectored by the tick species against which it is effective;
3. Provides long-lasting immunity;
4. Does not induce vaccine resistance (or evasion);
5. Does not induce adverse host responses (e.g., autoimmunity)
6. Cost-effective and practical

Interestingly, although subolesin fulfills many of these criteria, vaccination with subolesin reduced infection with several different bacterial and protozoan tick-borne pathogens but failed to protect against TBEV (Havlíková et al., 2013). Conversely, when 64TRP was used in a cattle tick trial to protect against *Theileria parva*, the protozoan tick-borne agent of East Coast fever in cattle, it was ineffective although 64TRP was effective against TBEV (Labuda et al., 2006; Olds et al., 2016). These contrasting results raise the question of whether a universal vaccine to protect against TBV, if achievable, may not provide similar protection against non-viral tick-borne pathogens. They point to a systems biology approach: we need to understand better the immunological environment at the site of tick feeding that prevents rather than ameliorates infection with TBVs and other tick-borne infectious agents. In the case of 64TRP, mice immunized with various constructs of the *R. appendiculatus*-derived saliva antigen showed significant levels of protection against lethal challenge by TBEV-infected *I. ricinus*, the natural virus vector that feeds on rodents at the immature stage (Labuda et al., 2006). When the surviving mice were inoculated with a lethal dose of TBEV, remarkably, they survived. Hence immunization with 64TRP created conditions at the tick feeding site that controlled the infection by tick bite in such a way that the tick-borne virus transmission effectively acted as a live attenuated anti-TBEV vaccine! This interpretation was supported by the results obtained when, in the same study, mice were immunized with either a licensed anti-TBEV vaccine or anti-tick (TickGARD) vaccine. Although the anti-TBEV vaccine gave slightly better protection against TBEV than 64TRP, it did not reduce significantly the number of mice supporting co-feeding TBEV transmission whereas 64TRP and TickGARD did. This result indicates that 64TRP and TickGARD induced a host response that interfered with virus uptake from the bloodmeal, possibly though antibody-mediated damage to the midgut, which of course would not occur in mice immunized against the virus. Significantly, although TickGARD reduced virus transmission (measured by the number of uninfected co-feeding nymphs that became infected) and number of mice supporting virus transmission, it did not protect mice against lethal infection with TBEV, in contrast to 64TRP. This strongly supports the hypothesis that inflammatory/immune response to antigenically cross-reactive secreted cement protein at the site of tick feeding on 64TRP-immunized mice (which did not occur in the immune response to the antigenically cross-reactive Bm86 midgut antigen) was responsible for the remarkable protective effect of the 64TRP vaccine. The nature of this host response was not determined although it appeared to be a predominantly CD8+ T lymphocyte response.

The surprising results obtained with 64TRP immunization highlight how little we understand the host responses that control tick infestations and TBV infections. By placing greater emphasis on tick-host-virus interactions as one “interactome” (rather than three separate interactions), we should move closer to specifying the ingredients required to generate an anti-tick vaccine that controls TBVs. Defining an environment at the tick-host interface that is “hostile” to TBVs opens up the possibility of creating a universal vaccine.

## Conclusions and future directions

Ticks succeeded in their role as blood-feeders and vectors of TBV thanks to their complex life history and feeding biology. Components in tick saliva have been found to play a crucial role in tick feeding and mediating transmission of TBV. Nowadays, an increasing body of evidence exists on (i) manipulation of host defenses by ticks to enhance feeding and promote pathogen transmission and (ii) strategies used by tick-borne pathogens to evade host immunity and ensure survival in different biological systems. However, much of this knowledge comes from tick and tick-borne bacteria interaction studies. Information on tick and TBV interaction is still limited and so far no tick molecules enhancing virus transmission have been identified. The systems biology approach employing transcriptomics and proteomics has started to reveal molecular mechanisms constituting the survival strategy and persistence of TBV in their vectors and vertebrate hosts as well as the interactions at the tick-virus-host interface determining virus transmission. Identification of SAT factors enhancing TBV transmission during the early phases of tick attachment in the host skin, but mainly the understanding of the complexity of the relationships between ticks, TBV, and their vertebrate hosts, will enable novel strategies for controlling ticks and viral tick-borne diseases.

## Author contributions

MK, PB, IS, ST, PN, MH, and VH conducted the literature search and wrote the paper. PB, PN, and MK prepared the figures and tables. All authors critically read and revised the manuscript.

### Conflict of interest statement

The authors declare that the research was conducted in the absence of any commercial or financial relationships that could be construed as a potential conflict of interest.
